# Self-assembly of nanoencapsulated undecanoic acid on cotton fiber for thermoregulating textiles

**DOI:** 10.1039/c9ra01602c

**Published:** 2019-03-29

**Authors:** Chunming Chen, Zhonghua Chen, Xingrong Zeng, Fei Yu

**Affiliations:** College of Chemistry and Environmental Engineering, Hanshan Normal University Chaozhou 521041 China; College of Materials Science and Engineering, South China University of Technology Guangzhou 510640 China cezhchen@scut.edu.cn +86-20-61088101 +86-20-61088108

## Abstract

We herein report the preparation of phase change nanocapsules with undecanoic acid (UA) as the core and a styrene–butyl acrylate copolymer as the shell by miniemulsion polymerization using interfacial redox initiation. The morphology, particle size, thermal properties, and structure of the nanocapsules were characterized by transmission electron microscopy, differential scanning calorimetry, and thermogravimetric analysis. Further, the cotton fiber was dissolved in hydroxyl-functionalized ionic liquid 1-(3-chloro-2-hydroxypropyl)-3-methyl imidazolium chloride, and then the nanoencapsulated UA was immobilized on the cotton fiber by mixing the solution with the phase change nanocapsule emulsion. The desired thermoregulating fabrics were then recovered by vacuum suction filtration and drying, and their surface morphologies and thermoregulating properties were evaluated. The nanocapsules were found to be of a regular spherical shape with a diameter ranging from 90 to 110 nm, and they exhibit a phase change temperature of 29.3 °C. Finally, the prepared thermoregulating non-woven fabrics exhibited excellent thermal reliability, air permeability, and thermoregulating capability.

## Abstract 2

We herein report the preparation of phase change nanocapsules with undecanoic acid (UA) as the core and a styrene–butyl acrylate copolymer as the shell by miniemulsion polymerization using interfacial redox initiation.

## Graphical abstract

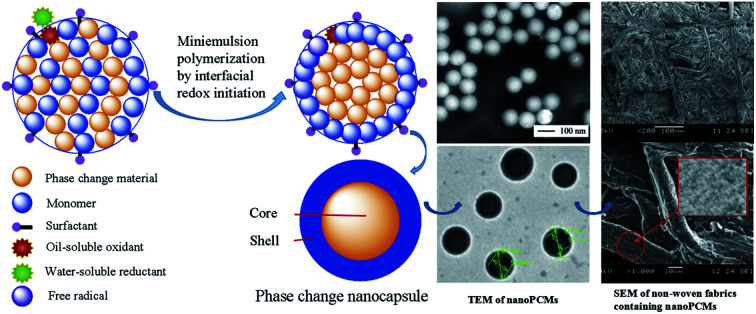

## Introduction

Thermoregulating fabrics are a kind of intelligent textile that provide an appropriate response to external temperature changes,^1,2^ where the degree of thermal comfort of the textile depends on the heat exchange between the human body and the surrounding environment. These fabrics can be developed by the incorporation of microencapsulated phase change materials (microPCMs) into textiles by spray coating and impregnation, or by direct incorporation into the fiber.^3–7^ Numerous studies have been carried out in this area in recent years, with examples including the incorporation of polystyrene microcapsules containing paraffin wax on a textile substrate to yield good thermoregulating properties,^8^ and investigation of the thermoregulating properties of a textile fabric containing melamine formaldehyde microcapsules with *n*-alkane mixtures and polyurethane to yield a coated fabric exhibiting good thermal and mechanical stability.^9^ Although microPCMs impart thermoregulating properties to the textile materials, high microPCM contents have adverse effects on the durability, moisture vapor permeability, elasticity, and softness of the coated fabrics.

Such encapsulated PCMs are generally classified as nanoencapsulated PCMs (nanoPCMs) and microPCMs according to their diameter (*i.e.*, 1–1000 μm for microPCMs and <1 μm for nanoPCMs).^10^ Recently, the preparation of nanoPCMs and their incorporation into textiles has received significant attention because of their narrow particle size and enhanced area of heat transfer. For example, Karthikeyan *et al.* developed a thermoregulating cotton fabric using nanoPCMs with an average diameter of 256 nm, which contain a paraffin wax core and urea–formaldehyde as the shell material, and gives a latent heat energy storage capacity of 1.91 J g^−1^ for the fabric containing 40 wt% nanocapsules.^11^ In addition, Choi *et al.* prepared textiles treated with nanoPCMs having an irregular size distribution of 200–400 nm, and evaluated their suitability as clothing materials to prepare thermostatic clothes. Interestingly, the air permeability, water vapor transmission, and the physical and mechanical properties of these treated fabrics were superior to those of fabrics treated with microPCMs.^12^ Furthermore, Sun *et al.* developed nanoPCMs containing paraffin PCMs for application on cotton fabric and polypropylene monofilaments.^2^ They found that nanoPCMs showed a superior durability on cotton fabric than microPCMs, and that a greater decrease in the latent heat was observed after washing in the case of fabric treated with microPCMs.^13–16^

**Fig. 1:**
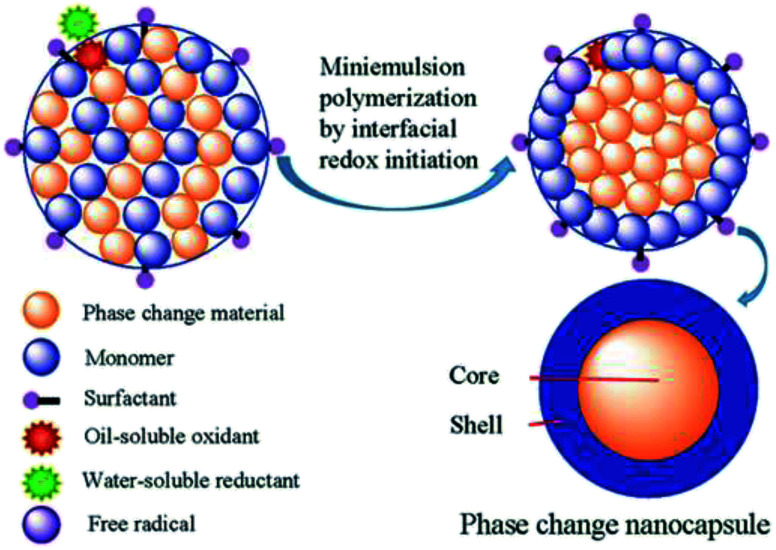
Schematic representation of the combination of miniemulsion polymerization with interfacial redox initiation.

For the development of nanoPCM technologies, various methods have been reported for the encapsulation of the PCMs, such as miniemulsion polymerization, interfacial polymerization, suspension cross-linking, and *in situ* polymerization.^17–19^ Among these methods, miniemulsion polymerization is a particularly attractive way to obtain nanocomposite particles with a uniform size. Indeed, we previously prepared *n*-octadecane nanocapsules with polystyrene shells using an ultrasonic-assisted redox interfacial initiation miniemulsion polymerization process.^20^ Redox interfacial initiation miniemulsion polymerization is an efficient method to control the location of free radical production and polymerization of the monomer. A schematic of the miniemulsion polymerization based on interfacial redox initiation is shown in Fig. 1. Using this process, a higher encapsulation efficiency can be obtained for the encapsulation of PCMs compared to that obtained by employing the thermal initiation system. The redox interfacial initiation system consists of a hydrophobic oxidant and a hydrophilic reductant; the hydrophobic oxidant would meet the hydrophilic reductant at the interface between the mini-droplets and the aqueous phase, thereby producing free radicals only at these locations. These radicals initiate the polymerization of the monomers, which is also confined to the interfaces between the mini-droplets and the aqueous phase.^21^ The polymer chains then grow gradually, thus forming of a polymer shell around the droplet, commonly yielding a high PCM encapsulation efficiency.^22^

Although nanoPCMs can improve the properties of textile materials, these properties depend on the nanoPCM loadings employed. To further enhance the thermoregulating performances of textiles, a high loading of nanoPCMs is therefore of crucial importance. Thus, we herein set out to develop thermoregulating fabrics through the incorporation of nanoPCMs in filament cotton fiber, and to improve the nanoPCM loading, and the durability and permeability of the treated textile. The raw cotton fibers will be dissolved in an ionic liquid (IL),^23^ and the resulting solution was mixed with a nanoPCM emulsion, followed by nanoPCM immobilization on the cotton fibers using a coagulation bath. The proposed thermoregulating fibers were then employed to fabricate thermoregulating non-woven fabrics by vacuum suction filtration and drying, and the resulting properties of the fabrics were evaluated.

## Experimental

### Materials

Undecanoic acid (UA, 99 wt%) was used as the core material, while styrene (St, 99.5 wt%), methacrylic acid (MA, 99.5 wt%), and *n*-butyl acrylate (BA, 98 wt%) were used as the shell-forming monomers. Cotton wool (100 wt%) was employed as the substrate, and all the above raw materials were purchased from the Shanghai Aladdin Reagent Co., Ltd. (China). Alkylphenol ether sulfosuccinate sodium salt (OS, 98 wt%, Jiangsu Haian Petro Chemical Plant, China) was used as the emulsifier. Hexadecane (HD, 98 wt%) was used as the co-emulsifier, while benzoyl peroxide (BPO, AR) and sodium formaldehyde sulfoxylate (SFS, 98 wt%) were used as the initiators; these reagents were purchased from Sinopharm Chemical Reagent Co., Ltd. (China). The St and BA monomers were washed several times with a 5 wt% aqueous NaOH solution and deionized water to remove the inhibitor prior to use.

### Preparation of the UA-containing nanocapsules

The redox interfacial initiation miniemulsion polymerization was carried out according to a previously reported technique.^20^ More specifically, the oil phase containing St (10 g), BA (0.8 g), MA (0.2 g), the desired quantity of UA, HD (0.4 g), and BPO (0.24 g) were stirred for 30 min at ambient temperature. The aqueous phase consisted of the OS emulsifier (1.5 g) and water (80 g), and this mixture was stirred for 30 min at 40 °C. After this, the two phases were mixed and allowed to stir at 40 °C and 1500 rpm over 30 min to obtain a pre-emulsion. This pre-emulsion was then treated using an ultrasonic homogenizer for 10 min in an ice-water bath to produce the miniemulsion, which was subsequently transferred to a 250 mL four-necked flask equipped with a mechanical stirrer and a reflux condenser. An aqueous solution of SFS (10 g water and 0.13 g SFS) was injected into the miniemulsion using a syringe pump over 1 h at 50 °C. Prior to the redox initiation, oil-soluble BPO molecules exist in the oil phase, and are mixed with the monomer and co-emulsion, while the water-soluble SFS molecules are present only in the aqueous phase. At the interfaces of the miniemulsion droplets, oil-soluble BPO molecules encounter the water-soluble SFS, which results in the production of free radicals to initiate monomer polymerization. The polymerization process was then carried out under a nitrogen atmosphere over 24 h at 45 °C, and the resulting emulsion was used for preparing the thermoregulating non-woven fabrics.

### Preparation of the thermoregulating fabrics

A functionalized IL containing an appended 3-chloro-2-hydroxypropyl group, namely 1-(3-chloro-2-hydroxypropyl)-3-methyl imidazolium chloride, was used as the solvent for the preparation of the thermoregulating fiber, and this IL was prepared according to our previous work.^24^ More specifically, ethanol (100 mL) and 0.2 mol hydrochloric acid were placed in a flask equipped with a reflux condenser and a magnetic stir bar, and *N*-methyl imidazole (0.2 mol) was added to the flask over 45 min at pH 6–6.5. After this time, epichlorohydrin (0.22 mol) was added dropwise to the solution, and the reaction was allowed to proceed for 3 h at 45 °C. 1-(3-Chloro-2-hydroxypropyl)-3-methyl imidazolium chloride was obtained by the removal of water, ethanol, and a small amount of epichlorohydrin by means of a reduced pressure distillation. Owing to its high polarity and hydroxyl-rich microenvironment, it is possible to prepare a cellulose solution of ≤10 wt%. Thus, the required quantity of cotton wool was added with the IL (10 mL) into a conical flask, and the mixture was heated at 60 °C with stirring at 200 rpm until dissolution of the cotton was complete and a transparent gel was obtained. The above-described nanoPCM-containing emulsion was then added to the conical flask, and the mixture was further stirred at 400 rpm for 30 min. Then, the nanoencapsulated UA was immobilized on the single cotton fiber by the self-assembly method, because of the abundant carboxylic acid groups on the surface nanoPCMs. Next, 50 mL of water was added and a white substance was precipitated using the coagulation bath method. The obtained precipitate was filtered by vacuum suction, washed with water, and the obtained membrane with a thickness of ∼0.01 mm was dried in a vacuum oven at 45 °C for 12 h. Finally, the surface morphology and thermoregulating properties of the obtained thermoregulating non-woven fabrics were evaluated. The IL was recycled and concentrated for reuse.

### Characterization

The thermal properties of the phase change nanocapsules were evaluated using a Netzsch DSC 204F differential scanning calorimeter (Germany) at a heating rate of 10 °C min^−1^ between 10 and 60 °C under a nitrogen atmosphere. The phase change latent heat and phase change temperature of the nanocapsules were determined from the DSC curves recorded using a Netzsch TA4 instrument.

The encapsulation efficiency can be calculated from the following equation:

1 *η* = (Δ*E*_n_/Δ*E*_o_) × (*m*_f_/*m*_o_) × 100%

where *η* is the encapsulation efficiency, Δ*E*_n_ is the phase change enthalpy of the nanocapsules, Δ*E*_o_ is the phase change enthalpy of pure UA (*i.e.*, 210.9 J g^−1^, from DSC measurements), *m*_o_ is the weight of UA, and *m*_f_ is the weight of UA plus the monomer.

Transmission electron microscopy (TEM) images of the nanocapsules were obtained using a HITACHI H-7650 transmission electron microscope (Japan) at an accelerating voltage of 80 kV. Scanning electron microscopy (SEM) images were obtained using a LEO 1530 VP Field Emission (FE) SEM instrument (Zeiss, Germany). The fiber samples were sputter-coated with a thin layer of gold in a vacuum chamber prior to SEM imaging.

The thermal stabilities of the dried nanocapsules were analyzed using a Netzsch TG 209F thermogravimeter (Germany) at a heating rate of 10 °C min^−1^ between 30 and 600 °C under a nitrogen atmosphere.

The air permeability of each textile was measured using an air-permeability tester (YG461E-II, Wuhan Guoliang Instrument Co., Ltd, China), and the air resistance values of the textile fabrics were determined under constant conditions for 24 h. The air resistance value (mm s^−1^) was taken as an average of ten sampling points, in accordance with ISO 9237.

**Fig. 2:**
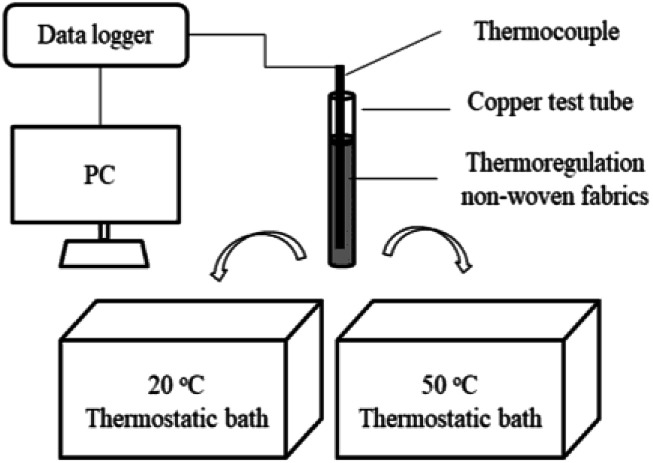
Schematic representation of the experimental apparatus employed for studying the thermal behavior of the thermoregulating non-woven fabrics.

The thermal storage and release performance tests were conducted using a constant temperature water bath. As shown in Fig. 2, the thermocouple was wrapped using the thermoregulating non-woven fabrics, and inserted into a copper test tube. This copper test tube was then placed in a hot water bath, and melting of the thermoregulating non-woven fabrics was carried out at a constant temperature of 50 °C. After completion of the melting process, solidification was carried out immediately by placing the copper test tube in cold water at a constant temperature of 20 °C. The temperature variations of the thermoregulating non-woven fabrics in the copper test tube were automatically recorded at 0.5 s intervals using a recording thermometer TWD-2 (Zhejiang, China) with an accuracy of ±0.1 °C. Each thermal cycling test involved one melting and freezing process, and this test was used to determine the thermal reliability of each of the prepared non-woven fabric.

## Results and discussion

### Effect of the monomer/UA mass ratio on the phase change enthalpy of the nanocapsules

**Fig. 3:**
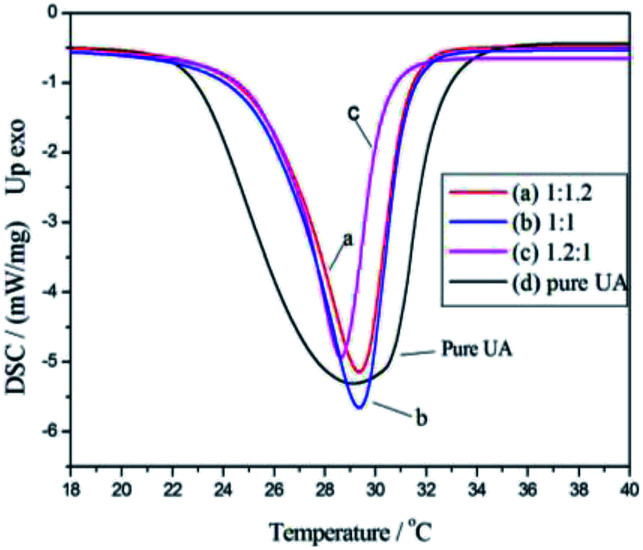
DSC curves of PCMs containing different monomer/UA mass ratios: (a) 1.2 : 1, (b) 1 : 1, (c) 1 : 1.2, and (d) UA.

To explore the effect of the monomer/UA mass ratio on the phase change enthalpy of the nanocapsules, a series of experiments using different monomer/UA mass ratios was carried out, and the obtained DSC curves are shown in Fig. 3, while the heat absorbing properties of the nanocapsules are listed in Table 1. As show, the nanocapsules exhibited a more endothermic peak than the pure St–BA–MA copolymer during heating between 20 and 50 °C. Furthermore, the highest nanocapsule encapsulation efficiency was obtained (99.3 wt%, calculated using eqn (1)) when a monomer/UA mass ratio of 1 : 1 was employed. These results indicate that the St–BA–MA copolymer could effectively encapsulate UA PCMs by miniemulsion polymerization using interfacial redox initiation. Moreover, the reduction in the phase change enthalpy of the UA-containing nanocapsules with monomer/UA mass ratios less than 1 : 1 is attributed to a shortage of copolymer, and therefore, the UA could not be completely covered. Based on these observations, a monomer/UA mass ratio of 1 : 1 was employed in subsequent polymerization reactions.

**Table 1:** Heat properties of the nanocapsules with different monomer/UA mass ratios

Monomer/UA mass ratio	Melting point (°C)	Melting enthalpy (J g^−1^)	Encapsulation efficiency (wt%)
1.2 : 1	29.2	99.4	98.6
1 : 1	29.3	105.1	99.3
1 : 1.2	29.3	108.5	98.3
UA	29.3	210.9	—

### Morphology of the UA-containing nanocapsules

**Fig. 4:**
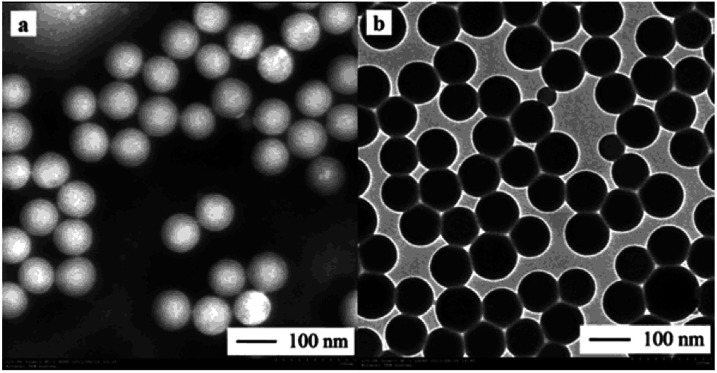
TEM images of the nanoPCMs. (a) A general view of a batch of nanoPCMs, and (b) diametrical view of the shell and core of the nanoPCMs upon staining with phosphotungstic acid at pH 2.0.

A TEM image of the nanocapsules is presented in Fig. 4. More specifically, Fig. 4a shows the general view of a batch of nanoPCMs, while Fig. 4b shows the diametrical view of the shell and core of the nanoPCMs following staining with phosphotungstic acid at pH 2.0. As shown, the nanocapsules particles have a uniform spherical shape with a distinct core–shell structure, and the UA core is located in the center of the St–BA–MA copolymer shell. The shell diameter ranges from 90 to 110 nm, and core diameter ranges from 60 to 90 nm. These results are consistent with those obtained using the particle size analyzer.

### TGA of the nanocapsules

**Fig. 5:**
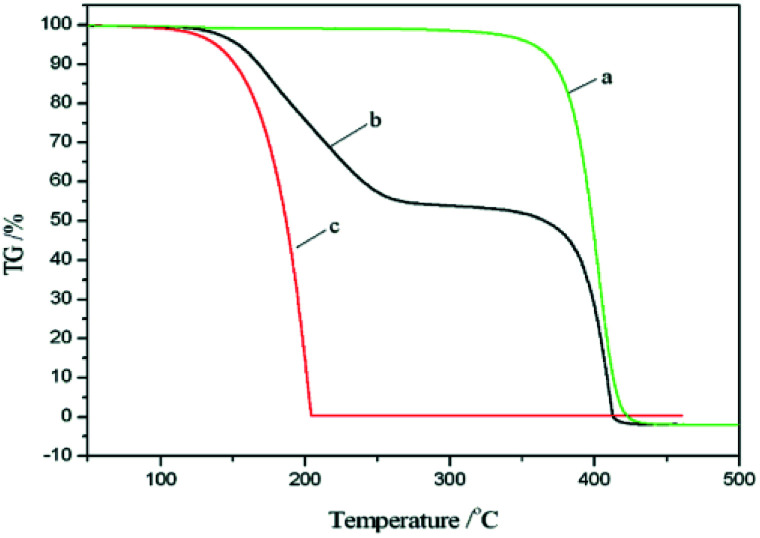
TG curves of (a) the shell materials, (b) the nanoPCMs, and (c) the pure UA.

Fig. 5 shows the TG curves of the shell material, the PCM nanocapsules, and pure UA. As shown in Fig. 5a, the mass loss process of the St–BA–MA copolymer shell material begins at ∼391.8 °C and ends at 430.5 °C. In addition, Fig. 5c shows that the mass loss from UA begins at ∼171.1 °C and ends at 207 °C, which in this case corresponds to the volatilization of UA. Furthermore, the onset temperature of mass loss for UA is significantly lower than that of the shell material, indicating that the thermal stability of the St–BA–MA copolymer shell is superior to that of pure UA. Moreover, as shown in Fig. 5b, the TG curve of the nanocapsules consists of two main stages, *i.e.*, 49.7% mass loss between 195.3 and 266.5 °C, corresponding to the gasification of UA, and 50.3% mass loss between 391.8 and 430.5 °C, corresponding to decomposition of the St–BA–MA copolymer. The onset temperature of the first mass loss step is higher in the case of the UA-containing nanocapsules compared to that of pure UA, indicating an improvement in the thermal stability of the phase change material following encapsulation.

### Morphology of the thermoregulating non-woven fabrics

**Fig. 6:**
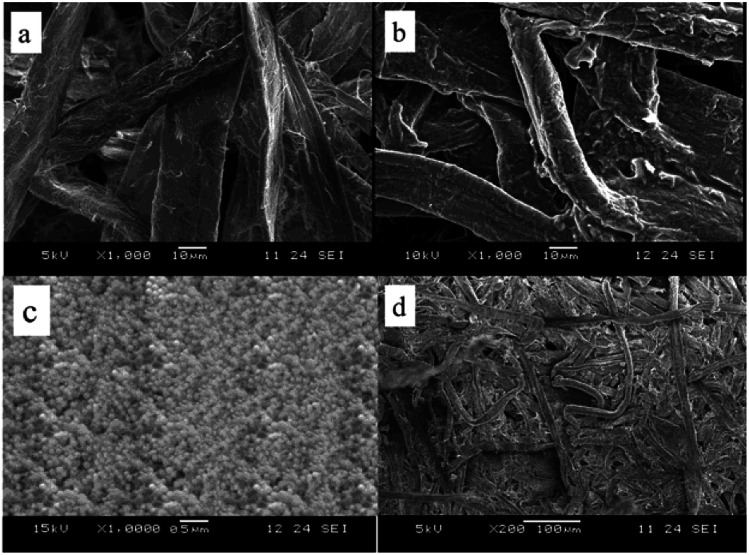
SEM images of (a) the raw cotton fiber, and (b) the nanoPCM-treated cotton fiber. (c) FE-SEM images of (c) the nanoPCM implants in the fiber, and (d) the non-woven fabrics containing the nanoPCMs.

The morphological structures of the fiber and the non-woven fabrics were also investigated through SEM observations (Fig. 6a–d). More specifically, Fig. 6a shows the relatively smooth surface of the untreated cotton fiber, while Fig. 6b shows that the nanoPCM-treated cotton fiber has a rough surface, thereby confirming the attachment of the PCM nanocapsules. In addition, Fig. 6c confirms the distribution of the PCM nanocapsules over the cotton fabric, where the nanocapsules surfaces are smooth and they are evenly distributed. Moreover, Fig. 6d shows the porous structure of the nanoPCM-containing non-woven fabrics, which implies that these materials can exhibit good thermoregulating performance and air permeability.

### The air permeability test

Air permeability is an important factor in determining the performance of a textile, and therefore, the air permeabilities of the prepared thermoregulating non-woven fabrics were determined according to ISO. As a reference, pure cotton spunlace nonwoven fabric (Jiaxing Furuisen Spunlaced Non-wovens Co., Ltd., China) was also tested under identical conditions. Thus, we determined the air resistance values of the thermoregulating non-woven fabrics and pure cotton spunlace nonwoven fabric to be 2032 and 2322 mm s^−1^, respectively (at equal thicknesses). These results indicate that the air permeability of the thermoregulating non-woven fabrics is lower than that of the cotton spunlace nonwoven fabric, likely due to the processing techniques involved. However, the air permeability of the fabric is only slightly lower, and it is therefore not considered particularly significant.

### Thermal storage and release performances of the thermoregulating non-woven fabrics

**Fig. 7:**
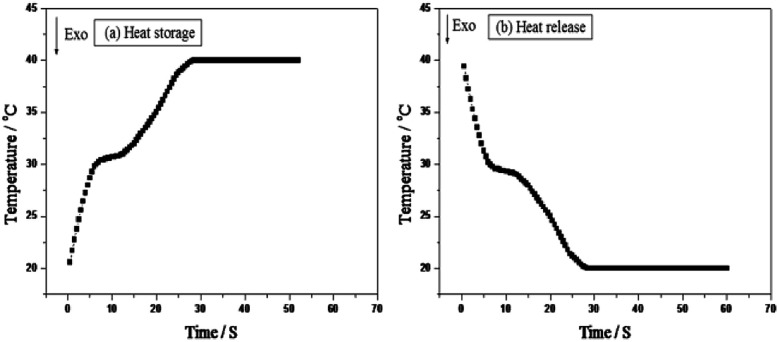
The heat storage/release performances of the thermoregulating non-woven fabrics.

Fig. 2 shows the experimental apparatus employed for evaluating the thermal storage and release performances of the thermoregulating nonwoven fabrics. The corresponding time–temperature melting and freezing curves are shown in Fig. 7. The thermoregulating non-woven fabrics exhibit a broad temperature range where a plateau is observed during melting and freezing, and the phase change temperatures of the UA nanocapsules can be determined. These results confirm that the prepared thermoregulating non-woven fabrics exhibit an excellent thermoregulation capability.

## Conclusions

We herein reported the successful preparation of nanocapsules of a phase change material (PCM) containing a styrene–*n*-butyl acrylate–methacrylic acid (St–BA–MA) copolymer as the shell and undecanoic acid (UA) as the core using the ultrasonic-assisted miniemulsion polymerization process, where the interfacial redox initiation of benzoyl peroxide (an oil-soluble oxidant) and sodium formaldehyde sulfoxylate (a water-soluble reductant) was employed instead of conventional thermal initiators. It was demonstrated that the interfacial redox initiation process promoted the nanoencapsulation of UA with high encapsulation efficiency. The obtained nanocapsules were spherical in shape with a distinct core–shell structure and 90–110 nm diameter. In addition, the UA-containing nanocapsules exhibited a phase change with latent heat of ∼105.1 J g^−1^ at 29.3 °C. Furthermore, for the preparation of the desired thermoregulating non-woven fabrics, cotton fiber was dissolved in an ionic liquid (*i.e.*, 1-(3-chloro-2-hydroxypropyl)-3-methyl imidazolium chloride), and mixed with the phase change nanocapsule emulsion, then the thermoregulating non-woven fabrics were obtained by vacuum suction filtration and drying. The obtained fabrics exhibited excellent thermal reliability, air permeability, and thermoregulation capability. These results are of importance because they demonstrate that a high loading of nanoPCMs on a textile material, can lead to enhanced thermoregulating performance.

## Conflicts of interest

There are no conflicts to declare.

## Supplementary Material
